# Radiotherapy plus temozolomide with or without anlotinib in H3K27M‐mutant diffuse midline glioma: A retrospective cohort study

**DOI:** 10.1111/cns.14730

**Published:** 2024-04-21

**Authors:** Chao Liu, Shuwen Kuang, Tianxiang Huang, Jun Wu, Longbo Zhang, Xuan Gong

**Affiliations:** ^1^ Department of Oncology Xiangya Hospital, Central South University Changsha China; ^2^ Department of Neurosurgery Xiangya Hospital, Central South University Changsha China

**Keywords:** adverse events, anlotinib, H3K27M mutant DMG, prognosis, survival, targeted therapy

## Abstract

**Background:**

Besides the hallmark of H3K27M mutation, aberrant amplifications of receptor tyrosine kinases (RTKs) are commonly observed in diffuse midline glioma (DMG), a highly malignant brain tumor with dismal prognosis. Here, we intended to evaluate the efficacy and safety of a multitarget RTK inhibitor anlotinib in patients with H3K27M‐DMG.

**Methods:**

A total of 40 newly diagnosed H3K27M‐DMG patients including 15 with anlotinib and 25 without anlotinib treatment were retrospectively enrolled in this cohort. Progression‐free survival (PFS), overall survival (OS), and toxicities were assessed and compared.

**Results:**

The median PFS and OS of all patients in this cohort were 8.5 months (95% CI, 6.5–11.3) and 15.5 months (95% CI, 12.6–17.1), respectively. According to the Response Assessment in Neuro‐Oncology (RANO) criteria, the disease control rate in the anlotinib group [93.3%, 95% confidence interval (CI), 70.2–98.8] was significantly higher than those without anlotinib (64%, 95% CI: 40.5–79.8, *p* = 0.039). The median PFS of patients with and without anlotinib was 11.6 months (95% CI, 7.8–14.3) and 6.4 months (95% CI, 4.3–10.3), respectively. Both the median PFS and OS of DMG patients treated with anlotinib were longer than those without anlotinib in the infratentorial patients (PFS: 10.3 vs. 5.4 months, *p* = 0.006; OS: 16.6 vs. 8.7 months, *p* = 0.016). Multivariate analysis also indicated anlotinib (HR: 0.243, 95% CI: 0.066–0.896, *p* = 0.034) was an independent prognosticator for longer OS in the infratentorial subgroup. In addition, the adverse events of anlotinib administration were tolerable in the whole cohort.

**Conclusions:**

This study first reported that anlotinib combined with Stupp regimen is a safe and feasible regimen for newly diagnosed patients with H3K27M‐DMG. Further, anlotinib showed significant efficacy for H3K27M‐DMG located in the infratentorial region.

## Graphical abstract

This retrospective cohort study investigated the tolerability and efficacy of Anlotinib combined with the Stupp regimen for patients with H3K27M mutant diffuse midline glioma (DMG). Beyond establishing the safety profile, our study also identified encouraging benefits associated with Anlotinib treatment, particularly for patients with infratentorial DMG. These findings suggest that Anlotinib may represent a promising treatment option for H3K27M mutant DMG in the clinical setting.

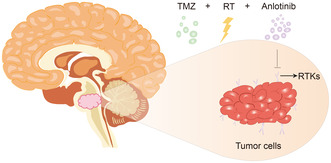

## 1 INTRODUCTION

H3K27M mutation was identified and associated with poor survival in diffuse intrinsic pontine glioma (DIPG).^1^ Thus, the new entity termed as “H3K27M mutant diffuse midline glioma (DMG)” has been proposed in the 2016 WHO classification of central nervous system (CNS) tumor, which integrated histopathology and molecular abnormalities.^2^ Previous studies indicated that H3K27M‐DMG predominantly occurs in children and adolescents and was relatively rarely found in adults.^3^ Owing to the high mortality, H3K27M‐DMG is the leading cause of CNS tumor‐related death in the pediatric population, accounting for about 10%–15% of all brain tumor deaths.^4^ Although comprehensive treatments containing surgical resection and chemoradiotherapy are adopted, the median overall survival (OS) of children with H3K27M‐DMG is only 9–12 months, with 2‐year survival of less than 10% since the time of diagnosis.^3^, ^5^ Moreover, several studies showed the prognosis differed significantly between tumors in different anatomical localizations. Infratentorial H3K27M‐DMG, particularly in the brainstem, showed worse survival than those located in the supratentorial region.^6^, ^7^

The conventional treatments of H3K27M‐DMG include surgical resection and chemoradiotherapy. Surgical resection is often restricted because of the high risk of neurological deficits. Due to the infiltrative and diffuse biological behavior, as well as anatomic adjacent of the critical brain eloquent areas such as brainstem and thalamus, total resection of H3K27M‐DMG is nearly impractical.^8^ Temozolomide (TMZ) is the mainly used chemotherapy for H3K27M‐DMG, even though most of the treated patients have TMZ resistance due to the intact blood–brain barrier (BBB) and the specific genetic phenotype of O6 methylguanine‐DNA methyltransferase (*MGMT*) promoter unmethylation.^9^ While radiotherapy offers temporary symptom relief and survival prolongation (70%–80% of patients),^10^, ^11^ tumor recurrence in the short term remains inevitable.

Recent studies focused on disclosing the epigenetic and genetic features of DMG to find novel therapeutic targets.^12^ The hallmark H3K27M mutation disrupts trimethylation at histone H3 lysine 27, leading to increased H3K27 acetylation and overexpression of genes involved in stem cell differentiation and tumorigenesis.^13^ Besides, aberrant amplification or mutation of receptor tyrosine kinases (RTKs) were also found in the H3K27M‐DMG. Among them, platelet‐derived growth factor receptor A (*PDGFRA*) amplification occurred in 32%–36% of DIPG, and epidermal growth factor receptor (*EGFR*) overexpression or amplification was present in 22.9%–27% of DMG.^14^ Previous studies also demonstrated that *PDGFRA* amplification occurred more frequently in brainstem tumors, while fibroblast growth factor receptor 1 (*FGFR1*) mutation was usually limited in the thalamus.^12^, ^15^ Both *PDGFR* inhibitor dasatinib and *FGFR* inhibitor panatinib exhibited antitumor effects in preclinical studies of DIPG.^16^, ^17^ However, a single RTK inhibitor had not shown satisfactory outcomes in DMG patients.^18^, ^19^ This is partially due to incomplete pathway network inhibition. Therefore, targeting multiple RTKs holds promise for enhanced antitumor efficacy in the treatment of H3K27M‐DMG.

Anlotinib is an oral multitargeted RTK inhibitor against tumor growth and angiogenesis by targeting *PDGFR*, *FGFR*, vascular‐endothelial growth factor receptor (*VEGFR*), *c‐kit*, and *Ret*.^20^ Currently, anlotinib has been approved by China National Medical Products Administration for the treatment of nonsmall‐cell lung cancer (NSCLC), small cell lung cancer, soft tissue sarcoma, and medullary thyroid cancer. In addition, anlotinib could cross the BBB confirmed by in vivo experiments and advanced NSCLC with brain metastasis.^21^ On the other hand, radiotherapy could increase the permeability of blood–brain barrier and increase anlotinib concentration in brain tissue. Thus, combining radiotherapy with anlotinib would benefit patients with brain metastases and gliomas.^22^, ^23^ Recently, several studies demonstrated anlotinib had favorable outcomes and tolerable adverse events (AEs) for recurrent high‐grade gliomas.^24^, ^25^ In our previous study, we also revealed the anlotinib combined with temozolomide could improve both progression‐free survival (PFS) and OS in the treatment of recurrent GBM with tolerant AEs.^26^ However, the efficacy of anlotinib treatment for H3K27M‐DMG has rarely been reported. So far, only two sporadic cases have been retrieved on the experimental clinical application of anlotinib.^27^, ^28^ Both the two patients achieved a satisfactory response with no serious AEs.

Based on the antiangiogenic and antitumoral effect of anlotinib as a multitargeted RTKs inhibitor as well as the frequently aberrant amplifications or mutation of RTKs in DMG, and previous favorable outcomes in two progressive DMG cases, we performed this cohort study to evaluate the efficacy and safety of anlotinib in patients with H3K27M‐DMG and also attempted to explore the genetic biomarkers of clinical benefits from anlotinib.

## 2 METHODS

### 2.1 Participants

Data of 40 patients with confirmed H3K27M‐DMG were retrospectively analyzed between July 2018 and November 2021 at Xiangya Hospital of Central South University. Based on the different treatment regimens, patients were divided into with anlotinib treatment or without anlotinib treatment and matched the baseline data. The inclusion criteria were as follows: (1) newly diagnosed intracranial DMG on the basis of pathologic diagnosis of H3K27M‐DMG according to the 2016 WHO classification of tumors of CNS (including tumors located at midline structures in the brain, tumor with histological characteristics of glioma, tumors with diffuse and infiltrative growth and H3K27M mutation confirmed both by immunohistochemistry (IHC) and/or Sanger sequencing); (2) newly diagnosed DMG with standard therapy, including surgical resection/stereotactic biopsy, concurrent chemoradiotherapy (CCRT), and adjuvant chemotherapy (AC) with TMZ according to Stupp regimen; (3) adequate organ function revealed by normal blood and urine routine, liver and kidney function, coagulation function, and electrocardiogram; (4) with complete follow‐up data, including regular Magnetic Resonance Imaging (MRI) evaluation, symptom changes, and cortisol administration. The exclusion criteria were as follows: (1) ganglioglioma or ependymoma with H3K27M mutation; (2) patients with relapsed DMG or treated with chemotherapy, radiotherapy, or target therapy before the confirmed diagnosis of H3K27M‐DMG; (3) with other malignant tumors or serious complications; (4) history of cerebral hemorrhage or infarction, stroke, transient ischemic attack, uncontrollable hypertension, history of myocardial infarction or unstable angina pectoris or arteriovenous thrombosis events within 6 months; (5) contraindication of TMZ, radiotherapy or anlotinib; (6) insufficient information on previous clinicopathological features or missing follow‐up data; (7) patients could not tolerate combined therapy or refuse. This study was approved by the Ethics Committee of Xiangya Hospital, Central South University (No. 202302015). All patients provided written informed consent and the patients' data were kept confidential.

### 2.2 Treatment

All the patients experienced the surgery, either cranial resection or stereotactic biopsy. Based on the extent of surgical resection, patients who experienced cranial resection were divided into gross total resection (GTR) (degree of resection 95%) and non‐GTR (degree of resection less than 95%). After surgery, all patients received intensity‐modulated radiotherapy (IMRT) with 54–60 Gy in 30 fractions, 1.8–2.0 Gy per day and 5 days a week for 6 weeks. According to Stupp regimen, concurrent chemotherapy TMZ (75 mg/m^2^/day) was orally administrated during IMRT. AC with TMZ was administrated after IMRT at most 6 cycles. The dose of a cycle was 150–200 mg/m^2^/day for 5 consecutive days, with 23 days of rest, and 28 days for a cycle of treatment. In anlotinib group, anlotinib was orally administrated 12 mg daily in 21‐day cycle (14 days on treatment from Day 1 to Day 14, 7 days on treatment from Day 15 to Day 21) initiated on the first day of radiation therapy and followed by adjuvant treatment with the same dosing schedule until patients have disease progression or intolerable toxicities as previous studies recommended.^26^, ^29^ Dosage would be reduced if intolerable grade 3 AEs occurred. Patients without anlotinib treatment received CCRT and AC only.

### 2.3 Efficacy evaluation and AEs

The efficacy was evaluated according to the Response Assessment for Neuro‐oncology (RANO) criteria.^30^ MRI were performed every 3 months or if symptoms worsened. Regular follow‐up visits were performed until disease progression or death. Disease status containing complete remission (CR), partial remission (PR), stable disease (SD), or progressive disease (PD) were assessed based on MRI and clinical symptoms. Disease control rate (DCR) was defined as the sum of PR, CR, and SD. PFS and OS were defined since the time from diagnosis (date of surgery). AEs were evaluated according to the National Cancer Institute‐Common Terminology Criteria Adverse Events version 4.0 (NCI‐CTCAE 4.0).

### 2.4 Statistical analysis

Statistical analyses were performed by GraphPad Prism 8 (GraphPad Software, La Jolla, CA, USA) and SPSS 23.0. The baseline and AEs data of patients were obtained by direct counting. PFS and OS were analyzed by Kaplan–Meier survival curves and compared by log‐rank test. Univariate and multivariate survival analyses were performed using the Cox proportional hazard model. Statistical analyses were performed using Student *t* test or Chi‐square (Fisher exact) test. *p* Value < 0.05 were defined as statistically significant.

## 3 RESULTS

### 3.1 General characteristics

A total of 40 newly diagnosed H3K27M‐DMG patients were included in this study. The baseline characteristics of all the patients are shown in Table 1. The median age was 17.5 years old (range, 4–65 years). There were 22 (55.0%) patients located in supratentorial and 18 (45.0%) patients located in infratentorial region. The median tumor size was 24.0 cm^3^. Most of patients (with anlotinib: 86.7%, without anlotinib: 84.0%) had a single lesion. Thirty‐six (90.0%) patients received surgery resection including GTR (27.5%) and non‐GTR (62.5%) in the whole cohort. Only one (4.0%) patient had IDH mutant in the group without anlotinib and one (6.7%) patient had MGMT promotor methylation in the group with anlotinib. There were 10 (66.7%) patients in the group with anlotinib and 13 (52.0%) patients in the group without anlotinib exhibiting a Ki‐67 index above 20%. The incidence rates of ATRX loss were similar in two groups.

**TABLE 1:** The baseline characteristics of 40 *H3* ^K27M^‐mutant DMG patients with and without anlotinib stratified by tumor location.

Characteristics, *n* (%)	Total			Supratentorial			Infratentorial		
	With anlotinib	Without anlotinib	*p*	With anlotinib	Without anlotinib	*p*	With anlotinib	Without anlotinib	*p*
No. cases	15	25		9	13		6	12	
Age			0.327			0.157			0.688
<18	6 (40.0)	14 (56.0)		1 (11.1)	5 (38.5)		5 (83.3)	9 (75.0)	
≥18	9 (60.0)	11 (44.0)		8 (88.9)	8 (61.5)		1 (16.7)	3 (25.0)	
Sex			0.327			1.000			0.344
Male	9 (60.0)	11 (44.0)		6 (66.7)	8 (61.5)		3 (50.0)	3 (25.0)	
Female	6 (40.0)	14 (56.0)		3 (33.3)	5 (38.5)		3 (50.0)	9 (75.0)	
KPS			0.444			0.544			1.000
<70	4 (26.7)	4 (16.0)		2 (22.2)	1 (7.7)		2 (33.3)	3 (25.0)	
≥70	11 (73.3)	21 (84.0)		7 (77.8)	12 (92.3)		4 (66.7)	9 (75.0)	
Tumor location			0.529			0.417			−
Thalamus	8 (53.3)	11 (44.0)		8 (88.9)	11 (84.6)		0	0	
Corpus callosum	0 (0)	1 (4.0)		0 (0)	1 (7.7)		0	0	
Basal ganglia	0 (0)	1 (4.0)		0 (0)	1 (7.7)		0	0	
Brain stem	6 (40.0)	12 (48.0)		0 (0)	0 (0)		6 (100.0)	12 (100.0)	
Others	1 (6.7)	0 (0)		1 (11.1)	0 (0)		0	0	
Tumor size (cm^3^)^a^			0.505			0.674			0.321
Mean ± SD	24.0 ± 17.8	24.2 ± 24.4		28.2 ± 21.5	34.8 ± 29.6		17.7 ± 7.8	12.8 ± 8.3	
≤Median	8 (53.3)	16 (64.0)		6 (66.7)	7 (53.8)		2 (33.3)	8 (66.7)	
>Median	7 (46.7)	9 (36.0)		3 (33.3)	6 (46.2)		4 (66.7)	4 (33.3)	
Tumor number			0.733			0.660			1.000
Single lesion	13 (86.7)	21 (84.0)		7 (77.8)	10 (76.9)		6 (100.0)	11 (91.7)	
Multiple lesion	2 (13.3)	3 (12.0)		2 (22.2)	2 (15.4)		0 (0)	1 (8.3)	
LMD	0 (0.0)	1 (4.0)		0 (0)	1 (7.7)		0 (0)	0 (0)	
Extent of surgery			0.662			0.151			0.529
Non‐GTR	10 (66.7)	15 (60.0)		4 (44.5)	5 (38.5)		6 (100.0)	10 (83.3)	
GTR	3 (20.0)	8 (32.0)		3 (33.3)	8 (61.5)		0 (0)	0 (0)	
Biopsy	2 (13.3)	2 (8.0)		2 (22.2)	0 (0)		0 (0)	2 (16.7)	
Molecular alteration									
*IDH*			1.000						1.000
−	15 (100.0)	24 (96.0)		9 (100.0)	13 (100.0)		6 (100.0)	11 (91.7)	
+	0 (0.0)	1 (4.0)		0 (0.0)	0 (0.0)		0 (0.0)	1 (8.3)	
*MGMT*			0.375			0.409			
−	14 (93.3)	25 (100.0)		8 (88.9)	13 (100.0)		6 (100.0)	12 (100.0)	
+	1 (6.7)	0 (0.0)		1 (11.1)	0 (0.0)		0 (0.0)	0 (0)	
Ki‐67			0.364			0.203			1.000
<20	5 (33.3)	12 (48.0)		2 (22.2)	7 (53.8)		3 (50.0)	5 (41.7)	
≥20	10 (66.7)	13 (52.0)		7 (77.8)	6 (46.2)		3 (50.0)	7 (58.3)	
ATRX			1.000			1.000			1.000
−	3 (20.0)	4 (20.0)		3 (33.3)	3 (27.3)		0	1 (11.1)	
+	12 (80.0)	16 (80.0)		6 (66.7)	8 (72.7)		6 (100.0)	8 (88.9)	
PFS (months)			0.216			0.864			0.006
Mean ± SD	11.8 ± 4.9	9.8 ± 9.8		12.3 ± 4.9	13.9 + 12.1		11.1 ± 5.4	5.4 + 2.4	
Median	11.6	6.4		12.8	10.4		10.3	5.4	
OS (months)			0.172			0.820			0.016
Mean ± SD	18.1 ± 6.3	15.6 ± 11.2		19.6 ± 6.6	21.1 + 13.0		16.0 ± 5.7	9.6 + 4.1	
Median	17.1	12.9		18.9	18.4		16.6	8.7	

Abbreviations: ATRX, alpha‐thalassemia mental retardation X‐linked; GTR, gross total resection; IDH1/2, isocitrate dehydrogenase 1/2; KPS, Karnofsky performance status; LMD, leptomeningeal dissemination; MGMT, O(6)‐methylguanine‐DNA methyltransferase; OS, overall survival; PFS, progression‐free survival; SD, standard deviation.

^a^ The median tumor size of all patients was 24.0 cm^3^. The median tumor size of patients with supratentorial and infratentorial tumors were 24.0 and 13.5 cm^3^, respectively.

### 3.2 Survival data and prognostic factors in the whole cohort

**FIGURE 1:**
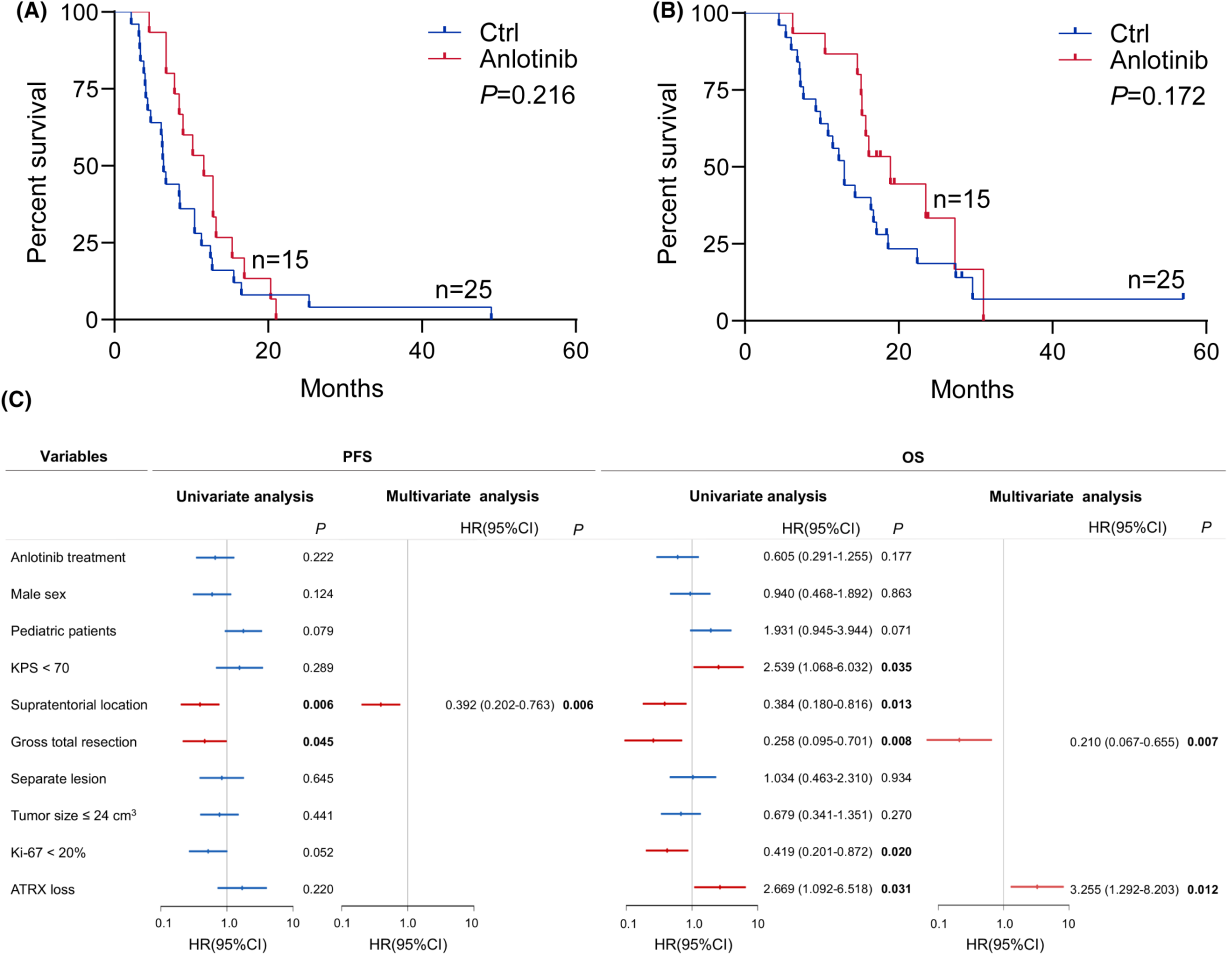
Kaplan–Meier curves of PFS (A) and OS (B) in the entire cohort. (C) Univariate and multivariate analyses of PFS and OS of all patients, based on treatment (with or without anlotinib), sex, age, KPS, tumor location (supratentorial or infratentorial region), extent of resection (GTR or non‐GTR/biopsy), tumor number (single lesion or multiple lesion), tumor size, and the expression of Ki‐67 and ATRX. GTR, gross total resection; KPS, Karnofsky performance status; OS, overall survival; PFS, progression‐free survival; ATRX, ATRX chromatin remodeler.

The median follow‐up time was 25.9 months (95% CI, 17.1–57.0). Until the follow‐up endpoint, 33 (82.5%) patients were dead. The median PFS and OS of all patients in this cohort were 8.5 months (95% CI, 6.5–11.3) and 15.5 months (95%CI, 12.6–17.1), respectively. According to RANO criteria, one patient achieved CR, three had PR, 10 experienced SD, and one developed PD in patients treated with anlotinib; while in the patients without anlotinib treatment, three patients reached CR, one patient had PR, 12 patients kept SD, and 9 developed PD. The disease control rate in anlotinib group (93.3%, 95% CI, 70.2–98.8) was significantly higher than that without anlotinib (64%, 95% CI, 40.5–79.8, *P* = 0.039). The median PFS of patients with and without anlotinib was 11.6 months (95% CI, 7.8–14.3) and 6.4 months (95% CI, 4.3–10.3), respectively. The median OS of patients with and without anlotinib was 17.1 months (95% CI, 15.2–21.5) and 12.9 months (95% CI, 9.2–16.9), respectively. Both median PFS and OS of patients with anlotinib were longer than those without anlotinib. However, the Kaplan–Meier survival curves and log‐rank tests showed no statistical significance in PFS (*p* = 0.216) or OS (*p* = 0.172) between the two groups (Figure 1A,B). Compared with the tumor located in the infratentorial region, supratentorial H3K27M‐DMG had a significantly better prognosis both in PFS (*p* = 0.006) and OS (*p* = 0.013). The patients experienced GTR had a better survival both in PFS (*p* = 0.045) an OS (*p* = 0.008) than those experienced non‐GTR or stereotactic biopsy. Besides, KPS ≥ 70 (*p* = 0.035), Ki‐67 < 20% (*p* = 0.020) and ATRX intact (*p* = 0.031) also indicated a longer OS. Multivariate survival analysis with the Cox proportional hazard model showed supratentorial lesion (HR: 0.392, 95% CI: 0.202–0.763, *p* = 0.006) was an independent prognostic factor for PFS, and GTR (HR: 0.210, 95% CI, 0.067–0.655, *p* = 0.007) and ATRX loss (HR, 3.255, 95% CI, 1.292–8.203, *p* = 0.012) were independent prognostic factors for OS (Figure 1C).

### 3.3 Anlotinib exhibited different efficacy in supratentorial and infratentorial DMG

**FIGURE 2:**
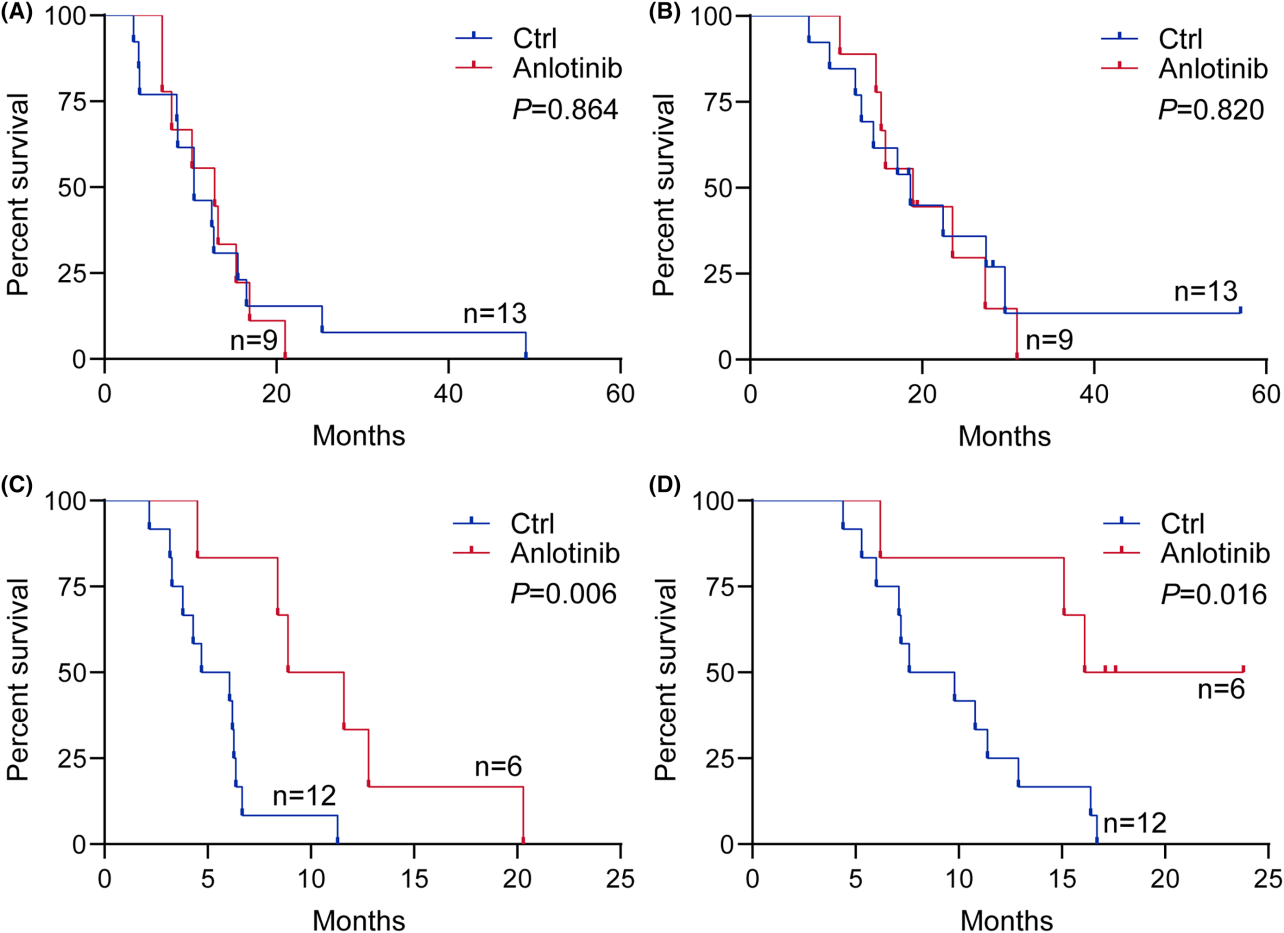
Kaplan–Meier curves of PFS (A) and OS (B) in the patients with supratentorial tumor, and PFS (C) and OS (D) in the patients with infratentorial tumor. PFS, progression‐free survival; OS, overall survival.

**FIGURE 3:**
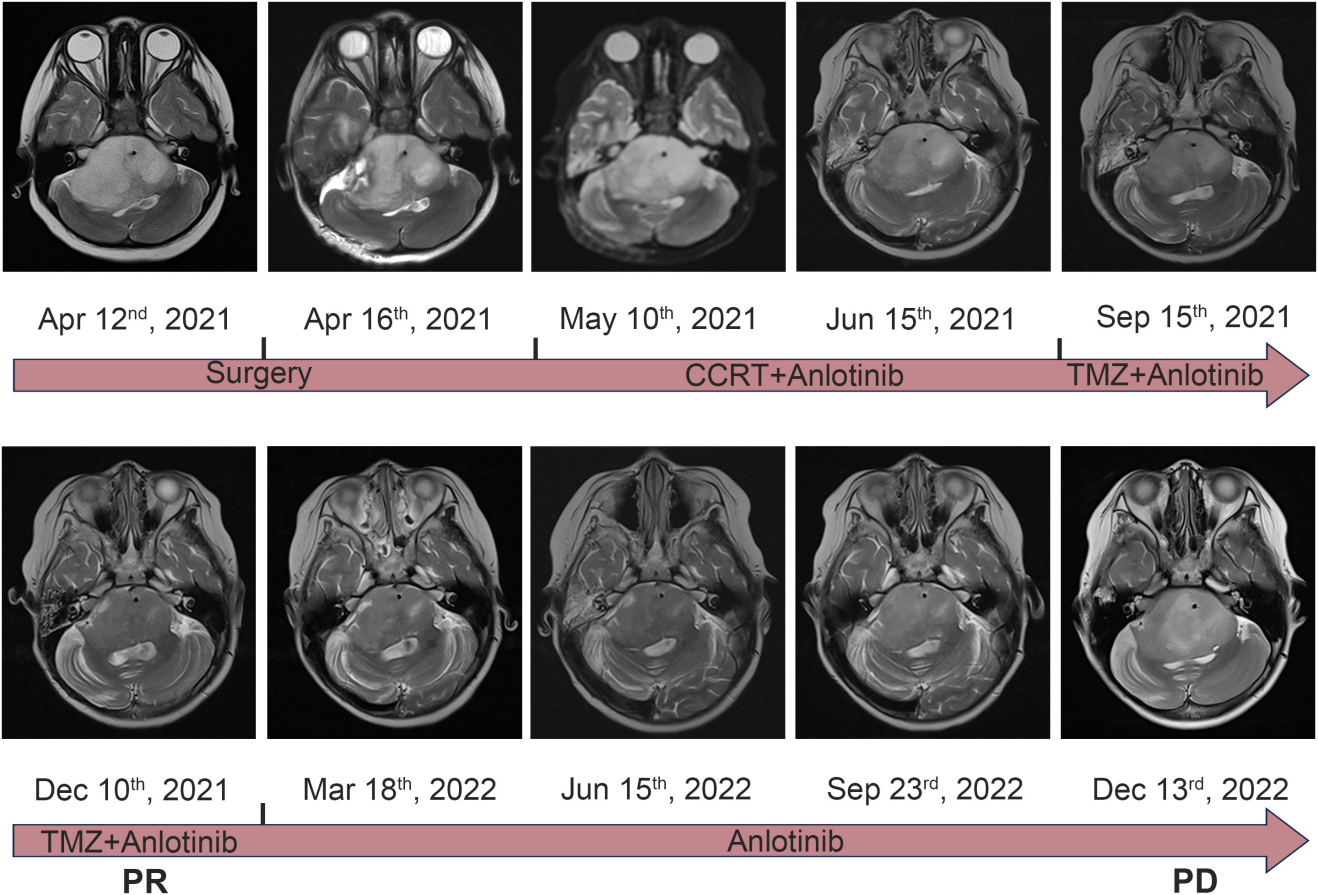
MRI changes of a representative case of DIPG treated with anlotinib. The best response achieved was PR, with a PFS of 20.3 months. CCRT, concurrent chemoradiotherapy; DMG, diffuse midline glioma; MRI, magnetic resonance imaging; PD, progressive disease; PR, partial remission; TMZ, temozolomide.

Several previous studies showed tumor location was related to the survival of H3K27M‐DMG and the prognosis of patients with infratentorial tumor was poorer than those with supratentorial tumor as well as different molecular alterations such as *PDGFRA* and *FGFR1* between brainstem and thalamus tumor.^31^, ^32^ It is possible that H3K27M‐DMG arising in different locations would have different responses to anlotinib. Then, we stratified H3K27M‐DMG by anatomic location to explore the efficacy of anlotinib treatment. In patients with supratentorial tumor, although the median PFS in the anlotinib group (12.8 months, 95% CI, 7.3–16.9) was longer than those without anlotinib (10.4 months, 95% CI, 8.4–15.5). However, there was no statistical difference in the PFS (*p* = 0.864) or OS (*p* = 0.820) between two groups in the supratentorial subgroup (Figure 2A,B). As for infratentorial tumor, both the median PFS and OS of patients with anlotinib treatment (PFS: 10.3 months, 95% CI, 4.5–16.6; OS, 16.6 months, 95% CI, 6.2–20.7) were longer than those without anlotinib (PFS: 5.4 months, 95% CI, 3.3–6.4; OS, 8.7 months, 95% CI, 6.0–12.9). The Kaplan–Meier survival curves and log‐rank tests showed the patients treated with anlotinib had significantly longer PFS (*p* = 0.006) and OS (*p* = 0.016) than those treated without anlotinib in infratentorial stratification (Figure 2C,D). Figure 3 shows MRI changes of an illustrative case with infratentorial tumor during treatments.

### 3.4 Prognostic factors in supratentorial and infratentorial H3K27M‐DMG

**FIGURE 4:**
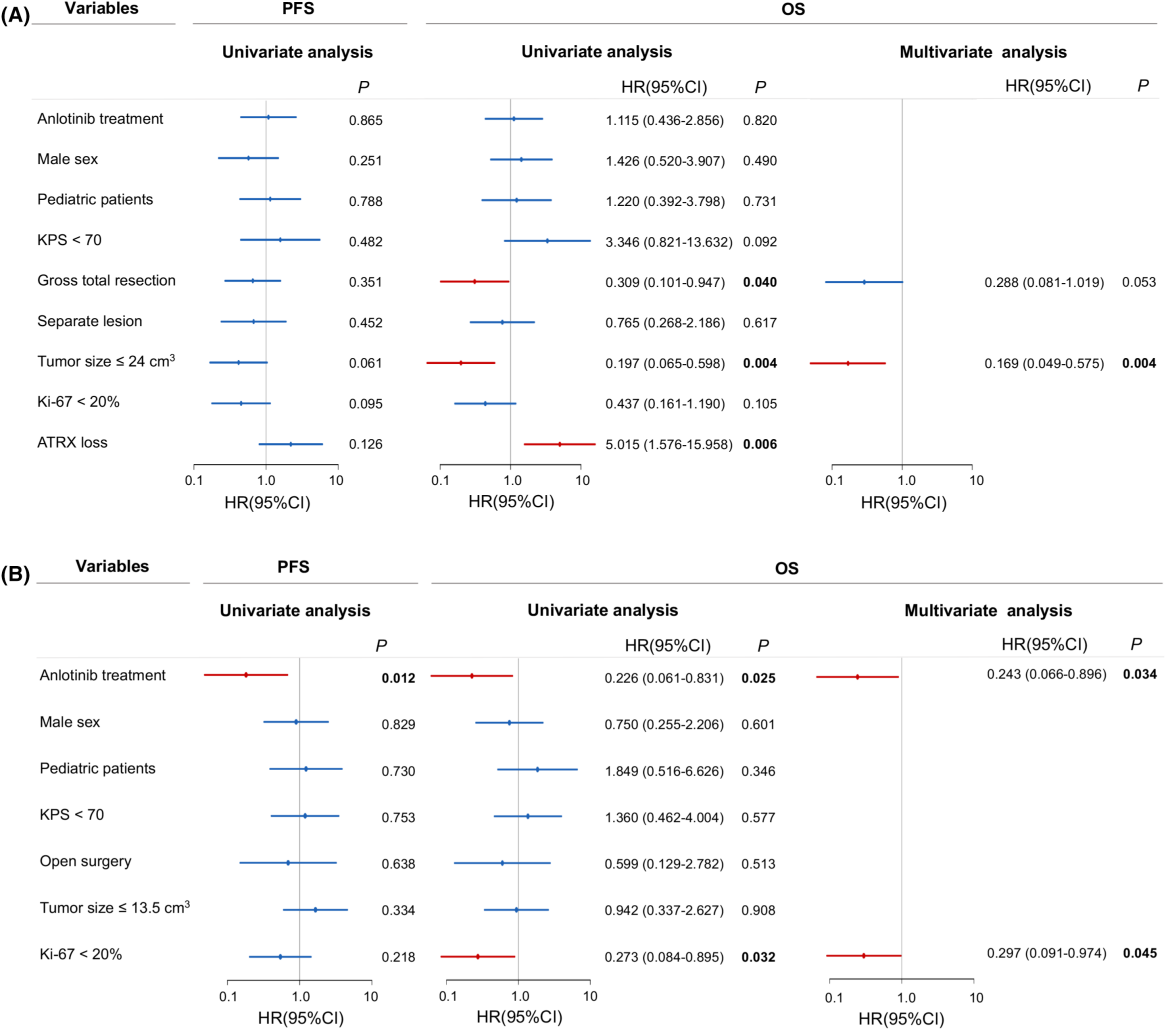
(A) Univariate and multivariate analyses of PFS and OS of patients with supratentorial tumor, based on treatment (with or without anlotinib), sex, age, KPS, extent of resection (GTR or non‐GTR/biopsy), tumor number (single lesion or multiple lesion), tumor size, and the expression of Ki‐67 and ATRX. (B) Univariate and multivariate analyses of PFS and OS of patients with infratentorial tumor, based on treatment (with or without anlotinib), sex, age, KPS, extent of resection (open surgery or biopsy), tumor number (single lesion or multiple lesions), tumor size, and the expression of Ki‐67. ATRX, ATRX chromatin remodeler; GTR, gross total resection; KPS, Karnofsky performance status; OS, overall survival; PFS, progression‐free survival.

The univariate and multivariate survival analyses were performed in supratentorial and infratentorial H3K27M‐DMG, respectively. In patients with supratentorial tumors, GTR (*p* = 0.040) and tumor size ≤24.0 cm^3^ (*p* = 0.004) indicated a better survival, while ATRX loss (*p* = 0.006) showed a worse prognosis. Cox survival analysis revealed tumor size ≤24.0 cm^3^ was the only significant prognosticator in supratentorial subgroup (Figure 4A). With regard to infratentorial tumors, anlotinib treatment was associated with longer PFS (*p* = 0.012) and OS (*p* = 0.025) and was a favorable independent factor for better OS (HR, 0.243, 95% CI, 0.066–0.896, *p* = 0.034). Moreover, Ki‐67 < 20% (HR, 0.297, 95% CI, 0.091–0.974, *p* = 0.045) was another positive prognosticator for longer OS (Figure 4B).

### 3.5 Genomic profiles of H3K27M‐DMG treated with anlotinib

**FIGURE 5:**
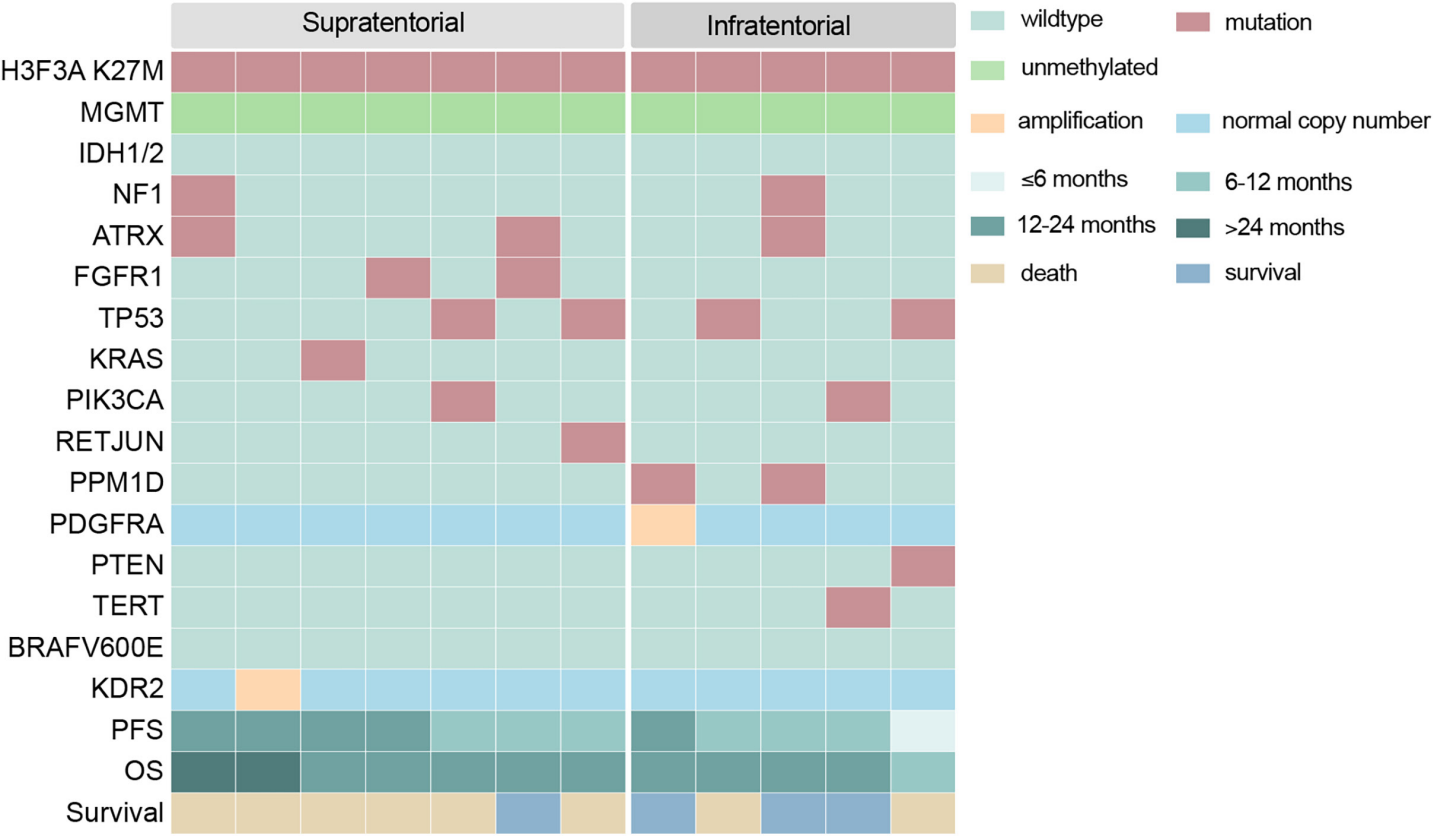
Molecular alterations of 12 *H3* ^K27M^‐mutant DMG patients undergoing anlotinib treatment with different tumor location tested by next‐generation sequencing. DMG, diffuse midline glioma.

To explore the relationship between genomic characteristics and survival, we performed the next‐generation sequencing in 12 patients treated with anlotinib (Figure 5). *TP53* mutation (4/12) was the most frequent alteration. RTK alterations were observed in four patients, including two *FGFR* mutations, one *PDGFRA* amplifications, and one *KDR2* amplification. In infratentorial subgroup, which was significantly responsive to anlotinib treatment, the patient harbored *PDGFRA* amplification had the best prognosis in both PFS and OS. And one with *PTEN* mutation had the poorest survival.

### 3.6 Compliance and toxicity

The comparison of AEs between patients with and without anlotinib is shown in Table 2. Compared with patients treating without anlotinib, hypertension (33.3% vs. 8.0%), hyperlipidemia (13.3% vs. 8.0%), thrombocytopenia (33.3% vs. 28.0%), and liver dysfunction (13.3% vs. 8.0%) were more common in patients administrated with anlotinib. The incidence rate of rash (13.3% vs. 12.0%) and leucopenia (20.0% vs. 20.0%) were similar in both two groups. Moreover, hand–foot skin reaction (HSFR) (26.7%), urine protein (26.7%), mouth ulcer (20.0%), hoarseness (6.7%), and hypothyroidism (6.7%) were exhibited only in the anlotinib group. Mild AEs could be tolerable after symptomatic treatment. Grade 3 AEs were reported in 33.3% of the patients receiving anlotinib. Among them, two patients had to reduce the dose to 10 mg/day due to foot blisters or thrombocytopenia. No patient discontinued treatment because of AEs and no grade 4 or higher toxicities were detected.

**TABLE 2:** The comparison of adverse events between patients with and without anlotinib.

Adverse events	With anlotinib		Without anlotinib	
	Grade 1/2 (*n*, %)	Grade 3 (*n*, %)	Grade 1/2 (*n*, %)	Grade 3 (*n*, %)
Hypertension	4/15 (26.7)	1/15 (6.7)	2/25 (8.0)	0
Hand‐foot skin reaction	3/15 (20.0)	1/15 (6.7)	0	0
Urine protein	3/15 (20.0)	1/15 (6.7)	0	0
Mouth ulcer	3/15 (20.0)	0	0	0
Hyperlipidemia	2/15 (13.3)	0	2/25 (8.0)	0
Hoarseness	1/15 (6.7)	0	0	0
Rash	2/15 (13.3)	0	3/25 (12.0)	0
Thrombocytopenia	3/15 (20.0)	2/15 (13.3)	5/25 (20.0)	2/25 (8.0)
Leucopenia	3/15 (20.0)	0	5/25 (20.0)	0
Liver dysfunction	2/15 (13.3)	0	2/25 (8.0)	0
Hypothyroidism	1/15 (6.7)	0	0	0

## 4 DISCUSSION

H3K27M‐DMG patients face a grim prognosis despite surgery and conventional chemoradiotherapy. Unfortunately, therapeutic options for this aggressive brain tumor remain limited. While surgical resection/biopsy is the most common initial treatment, it serves several purposes: confirming histological and molecular diagnosis for potential clinical trials, reducing the tumor burden, improving neurological function, and relieving hydrocephalus in some cases.^33^, ^34^

Maximum safe resection is recommended whenever feasible for newly diagnosed gliomas, including DMG.^2^, ^35^ Consistent with a recent systematic review of 484 H3K27M‐DMG patients showing resection as an independent favorable prognosticator,^6^ our study found GTR to be associated with better prognosis and longer PFS in this patient population. Notably, several studies determined resection was not a prognostic factor for DMG, highlighting the need for further investigation.^34^, ^36^ In our supratentorial tumor cohort, GTR was correlated with improved OS. However, the role of surgery for infratentorial DMG, primarily located in the pons where achieving GTR is still controversial. An increasing number of studies have also demonstrated the safety of stereotactic biopsy in H3K27M‐DMG.^37^, ^38^ In our study, the survival of two infratentorial DMG patients who underwent stereotactic biopsy was comparable to those who received surgical resection.

Recently, more and more clinical trials have increasingly focused on the genetic and epigenetic underpinnings of DMG to identify novel therapeutic targets (Table 3). Recent research has identified amplification or overexpression of several RTKs in DMG, including *PDGFRA*, *EGFR*, *VEGFR2*, *FGFR1*, and *Met*.^17^, ^39^, ^40^, ^41^ Inhibitors targeting RTKs and their downstream signaling pathways have exhibited promising antitumor effect in preclinical and clinical studies of for DMG and DIPG (See Table S1). Additionally, these inhibitors have been shown to enhance the sensitivity to radiation therapy.^42^ However, several early phase clinical trials for DIPG reported minimal efficacy of some RTK inhibitors.^43^, ^44^ This could be partly attribute to compensatory feature of RTK co‐activation networks, where inhibiting a single RTK pathway fails to address other related tumorigenesis‐promoting pathways.^45^ Multi‐targeted RTK inhibitors may improve upon the shortcomings of single‐agent therapy in DIPG patients.^46^

**TABLE 3:** Recent and ongoing clinical trials about targeted therapy in H3K27M‐mutant DMG or DIPG.

Targets	Intervention	Administration	Clinical trial	Tumor eligibility	Phase	Recruitment status
EGFR	Nimotuzumab, CRT	IV	NCT04532229	Newly diagnosed	III	Recruiting
PDGFRα, Kit	Avapritinib	PO	NCT04773782	Diagnosed	I/II	Recruiting
Multi‐kinase	Vandetanib, dasatinib	PO	NCT00996723	Newly diagnosed	I	Completed
	Cabozantinib	PO	NCT05135975	Recurrent, progressive	II	Recruiting
VEGF	Bevacizumab, RT	Injection	NCT04250064	Diagnosed	II	Recruiting
PI3K, mTOR	GDC‐0084, RT	PO	NCT03696355	Newly diagnosed	I	Completed
Wee1	Adavosertib, RT	PO	NCT019220766	Newly diagnosed	I	Completed
CDK4/6	Ribociclib, Everolimus	PO	NCT03355794	Newly diagnosed	I	Completed
	Abemaciclib	PO	NCT02644460	Diagnosed	I	Recruiting
HDAC	Panobinostat, Marizomib	PO	NCT04341311	Diagnosed	I	Active, not recruiting
HDAC/PI3K	Fimepinostat	PO	NCT03893487	Newly diagnosed	I	Active, not recruiting
DRD2	ONC206	PO	NCT04541082	Recurrent	I	Recruiting
	ONC206, RT	PO	NCT04732065	Newly diagnosed, recurrent	I	Recruiting
	ONC201	PO	NCT02525692	Recurrent	II	Active, not recruiting
	ONC201	PO	NCT03416530	Newly diagnosed, recurrent, refractory	I	Active, not recruiting
	ONC201	PO	NCT03295396	Recurrent	II	Active, not recruiting
	ONC201	PO	NCT05580562	Newly diagnosed	III	Recruiting
	ONC201, Everolimus, RT	PO	NCT05476939	Diagnosed	III	Recruiting
	ONC201, RT, Paxalisib	PO	NCT05009992	Newly diagnosed, recurrent, progressive	II	Recruiting
XPO1	Selinexor, RT	PO	NCT05099003	Newly‐diagnosed	I/II	Recruiting
ADAM	INCB7839	IV	NCT04295759	Recurrent, progressive	I	Recruiting
IDO1	Indoximod, RT	PO	NCT04049669	Newly diagnosed, relapsed	II	Recruiting
CD40	APX005M	IV	NCT03389802	Newly diagnosed, recurrent, refractory	I	Active, not recruiting

Abbreviations: ADAM, a disintegrin and metalloprotease; CRT, chemoradiotherapy; DIPG, diffuse intrinsic pontine glioma; DMG, diffuse midline glioma; DRD2, dopamine receptor D2; HDAC, histone deacetylase; Kit, Kit proto‐oncogene; IV, intravenous therapy; mTOR, mechanistic target of rapamycin; PDGFRα, platelet‐derived growth factor receptor α; PI3K, phosphatidylinositide 3‐kinases; PO, peros; RT, radiotherapy; VEGF, vascular‐endothelial growth factor; Wee1, Wee1 G2 checkpoint kinase; XPO1, exportin‐1; IDO1, indoleamine 2,3‐dioxygenase‐1; CDK4/6, cyclin dependent kinase 4/6.

Anlotinib is small molecule RTKs inhibitor with a broad spectrum. Previous studies reported favorable responses in NSCLC patients with brain metastases, demonstrating its ability to control brain tumors and survival.^22^, ^47^ Furthermore, Ma et al.^21^ confirmed optimal penetration of anlotinib into brain microvascular endothelial cells, potentially overcoming the limited drug concentration in brain tumors due to the blood–brain barrier (BBB). These findings suggest that anlotinib is a promising candidate for treating intracranial malignant tumors. Our previous study demonstrated the safety and efficacy of combining anlotinib with dose‐dense TMZ for recurrent GBM,^26^ consistent with other research.^24^, ^48^ While only two case reports documented the benefit of anlotinib in progressive H3K27M‐DMG.^27^, ^28^

Our study found that patients treated with anlotinib had higher rates of DCR and longer PFS and OS compared to those who did not receive anlotinib, even though the increase in PFS and OS did not reach statistical differences in the entire cohort. Notably, when stratified by tumor location, our findings demonstrated a significantly more favorable response to anlotinib treatment in patients with infratentorial tumors compared to their supratentorial counterparts in terms of PFS and OS. Moreover, multivariate analysis identified anlotinib treatment as an independent favorable prognosticator for patients with infratentorial DMG. The median PFS and OS for infratentorial tumor patients treated with anlotinib were 10.3 months (95% CI, 4.5–16.6) and 16.6 months (95% CI, 6.2–20.7), respectively, exceeding outcomes reported in the literature for Stupp regimen used in DIPG.^49^ Compared to our study, targeted therapies such as nimotuzumab (mPFS: 5.8 months, mOS: 9.4 months) and bevacizumab (mOS: 10.4 months) showed modest benefits in DIPG.^50^, ^51^ A recent small molecule selective antagonist of dopamine receptor D2/3 (DRD2/3) ONC201 has emerged another treatment option for H3K27M‐DMG, with a reported median OS of 21.7 months in the patients including infratentorial and supratentorial DMG.^52^ While both anlotinib and ONC201 demonstrate promising antitumor activity in H3K27M‐DMG patients, our data suggest that infratentorial DMG patients may benefit more from anlotinib treatment. The distinct tumor response to anlotinib observed in different anatomical localization may be associated with underlying molecular alterations. Previous studies indicated that *PDGFRα* amplification was frequently observed in DIPG or brainstem tumor.^12^, ^14^, ^15^, ^39^ In our study, the patient with infratentorial DMG who harbored *PDGFRα* amplification exhibited the best response to anlotinib. Due to the limited sample size, we cannot determine whether *PDGFRα* amplification is the genetic biomarker predicting benefit from anlotinib treatment. Further studies with larger cohorts are warranted to explore this possibility.

Anlotinib treatment for DMG appears to be well tolerated with manageable side effects. Our study observed mostly Grade 1 or 2 AEs, with no instances of Grade 4 AEs or anlotinib‐related deaths. The rate of Grade 3 AEs was 33.3% in patients receiving anlotinib, which compares favorably to ONC201 (41.7%).^53^ Thrombocytopenia is a common side effect in our study. It is important to note that a separate retrospective analysis suggests this might be associated with the combination of anlotinib and TMZ, rather than anlotinib alone.^48^ Our results align with previous studies highlighting hypertension, proteinuria, and HSFR were the most frequent anlotinib‐associated side effects.^20^, ^29^ Fortunately, anlotinib‐induced AEs could be managed through dosage adjustments.^54^ In our cohort, two patients required dose reduction due to severe grade 3 AEs. However, dose reduction and drug discontinuation due to AEs can negatively impact treatment compliance and potentially compromise therapeutic benefits, as reported in advanced NSCLC and metastatic colorectal cancer.^55^, ^56^ Thus, further research is necessary to determine an optimal dosage regimen for anlotinib treatment in H3K27M‐DMG patients.

This study has several limitations. First, this is a retrospective study with limited sample size. Although baseline characteristics were balanced between the anlotinib and control groups to minimize bias, the possibility of selection bias cannot be entirely ruled out. Second, the sample size in this study was limited. Large randomized controlled trials and large‐scale genetic sequencing are warranted to provide more robust evidence for the efficacy and potential biomarkers of anlotinib in DMG treatment.

## 5 CONCLUSION

To our knowledge, this is the first retrospective cohort study to investigate the use of anlotinib combined with the Stupp protocol for H3K27M‐DMG treatment. Our findings suggest that this combination therapy appears to be a safe and promising therapeutic option for this patient population. Further, anlotinib exhibited inspiring survival benefits for infratentorial H3K27M DMG patients.

## AUTHOR CONTRIBUTIONS

Chao Liu: Collection and/or assembly of data, Manuscript writing. Shuwen Kuang: Collection and/or assembly of data, Manuscript writing. Tianxiang Huang: Critical revision of the manuscript. Jun Wu: Critical revision of the manuscript. Longbo Zhang: Critical revision of the manuscript. Xuan Gong: Conception/design, Supervision, Collection and/or assembly of data, Manuscript writing—review and editing.

## FUNDING INFORMATION

This study was supported by the National Natural Science Foundation of China (grant no. 81701285), the Nature Science Foundation of Hunan Province (grant no. 2018JJ3824), and the Nature Science Youth Foundation of Hunan Province (grant no. 2018JJ3856).

## CONFLICT OF INTEREST STATEMENT

The authors declare no conflict of interest.

## Supporting information


Appendix S1:

## Data Availability

The data supporting the findings of this article will be available from the corresponding authors upon on reasonable request.
