# Supplementary material for: Radiotherapy plus temozolomide with or without anlotinib in H3K27M‐mutant diffuse midline glioma: A retrospective cohort study
Source: CNS Neurosci Ther. 2024 Apr 21;30(4):e14730. doi: 10.1111/cns.14730 (PMC11033330; doi:10.1111/cns.14730)
Supplement: Supplementary file 1 — Appendix S1: [file CNS-30-e14730-s001.docx]

**Supplementary table 1** RTKs and related pathway inhibitors used in preclinical or clinical therapies for DMG or DIPG

| Targets | Drugs | Observations | References |
| --- | --- | --- | --- |
| PDGFR | Crenolanib | The OS was similar to historical controls in Phase I DIPG trial | ^1^ |
|  | Dasatinib | Inhibited DIPG growth combining with cabozantinib in vitro | ^2^ |
|  |  | Showed minimal activity and poor toleration combing with c met inhibitor crizotinib in Phase I pediatric DIPG trial | ^3^ |
|  |  | Showed antitumor effect with trametinib in DIPG in vivo and in vitro | ^4^ |
|  | Imatinib | Showed slightly objective response with RT in newly diagnosed brainstem gliomas | ^5^ |
| EGFR | Erlotinib | Combined with bevacizumab and irinotecan prolonging the OS in phase I/II DIPG trial, compared with previous literatures | ^6^ |
|  | Gefitinib | Showed little favorable outcome combing with RT in phase II newly-diagnosed pediatric diffuse intrinsic brainstem glioma trial | ^7^ |
|  | Cetuximab | The OS was longer than historic control when combining with bevacizumab in Phase I DIPG trial | ^8^ |
|  |  | No improvement in the poor survival time with RT in pediatric DIPG trial | ^9^ |
|  | Nimotuzumab | Modest activity in phase II progressive DIPG trial | ^10^ |
|  |  | The PFS and OS were similar to historical controls in the condition of combing with RT in Phase III newly diagnosed DIPG trial | ^11^ |
| FGFR | Ponatinib | Showed antiproliferative effect in DIPG cells | ^12^ |
| c met | Crizotinib | No antitumor activity with dasatinib in in Phase I progressive/recurrent pediatric DIPG trial | ^13^ |
| Multi-kinases | Anlotinib | Case showed reduced tumor size and prolonged survival time combing with TMZ in *H3*^K27M^ mutant DMG | ^14^ |
|  |  | Case showed prolonged OS in *H3*^K27M^ mutant DMG with *PDGFRα* mutation | ^15^ |
|  | Vandetanib | No change in the poor prognosis with dasatinib in Phase I DIPG trial | ^16^ |
|  |  | Controlled disease progression combing with RT in Phase I pediatric DIPG trial | ^17^ |
|  |  | Cases showed slight effect with everolimus in ACVR1 mutant pediatric DIPG | ^18^ |
| PI3K/AKT | Perifosine | Modest activity in usage alone and provided no additional survival benefit to high dose RT in brainstem glioma model | ^19^ |
| MEK/ERK | Trametinib | Synergistic antitumor effects with PI3K/AKT inhibitor perifosine in DIPG Cells | ^20^ |
| mTOR | TAK228 | Inhibited tumorigenicity and enhances radiosensitization in DIPG in vivo and in vitro | ^21^ |
|  | AZD2014 | Inhibited DIPG growth in vitro | ^22^ |

Abbreviations: DMG, diffuse midline glioma; DIPG, diffuse intrinsic pontine glioma; PDGFR, platelet-derived growth factor receptor; OS, overall survival; c-Met, Met proto-oncogene; RT, radiotherapy; EGFR, endothelial growth factor receptor; FGFR, fibroblast growth factor receptor; PFS, progression-free survival; ACVR1, activin A receptor type 1; HGG, high-grade glioma; PI3K, phosphatidylinositide 3-kinases; AKT, AKT serine/threonine kinase; MEK/ERK, mitogen-activated protein kinase(MAPK); mTOR, mechanistic target of rapamycin.

**References**

1. Tinkle CL, Broniscer A, Chiang J, et al. Phase I study using crenolanib to target PDGFR kinase in children and young adults with newly diagnosed DIPG or recurrent high-grade glioma, including DIPG. *Neurooncol Adv.* 2021;3(1):vdab179.

2. Truffaux N, Philippe C, Paulsson J, et al. Preclinical evaluation of dasatinib alone and in combination with cabozantinib for the treatment of diffuse intrinsic pontine glioma. *Neuro Oncol.* 2015;17(7):953-964.

3. Broniscer A, Jia S, Mandrell B, et al. Phase 1 trial, pharmacokinetics, and pharmacodynamics of dasatinib combined with crizotinib in children with recurrent or progressive high-grade and diffuse intrinsic pontine glioma. *Pediatr Blood Cancer.* 2018;65(7):e27035.

4. Izquierdo E, Carvalho DM, Mackay A, et al. DIPG Harbors Alterations Targetable by MEK Inhibitors, with Acquired Resistance Mechanisms Overcome by Combinatorial Inhibition. *Cancer Discov.* 2022;12(3):712-729.

5. Pollack IF, Jakacki RI, Blaney SM, et al. Phase I trial of imatinib in children with newly diagnosed brainstem and recurrent malignant gliomas: a Pediatric Brain Tumor Consortium report. *Neuro Oncol.* 2007;9(2):145-160.

6. El-Khouly FE, Veldhuijzen van Zanten SEM, Jansen MHA, et al. A phase I/II study of bevacizumab, irinotecan and erlotinib in children with progressive diffuse intrinsic pontine glioma. *J Neurooncol.* 2021;153(2):263-271.

7. Pollack IF, Stewart CF, Kocak M, et al. A phase II study of gefitinib and irradiation in children with newly diagnosed brainstem gliomas: a report from the Pediatric Brain Tumor Consortium. *Neuro Oncol.* 2011;13(3):290-297.

8. McCrea HJ, Ivanidze J, O'Connor A, et al. Intraarterial delivery of bevacizumab and cetuximab utilizing blood-brain barrier disruption in children with high-grade glioma and diffuse intrinsic pontine glioma: results of a phase I trial. *J Neurosurg Pediatr.* 2021;28(4):371-379.

9. Macy ME, Kieran MW, Chi SN, et al. A pediatric trial of radiation/cetuximab followed by irinotecan/cetuximab in newly diagnosed diffuse pontine gliomas and high-grade astrocytomas: A Pediatric Oncology Experimental Therapeutics Investigators' Consortium study. *Pediatr Blood Cancer.* 2017;64(11):10.1002/pbc.26621.

10. Bartels U, Wolff J, Gore L, et al. Phase 2 study of safety and efficacy of nimotuzumab in pediatric patients with progressive diffuse intrinsic pontine glioma. *Neuro Oncol.* 2014;16(11):1554-1559.

11. Fleischhack G, Massimino M, Warmuth-Metz M, et al. Nimotuzumab and radiotherapy for treatment of newly diagnosed diffuse intrinsic pontine glioma (DIPG): a phase III clinical study. *J Neurooncol.* 2019;143(1):107-113.

12. Schramm K, Iskar M, Statz B, et al. DECIPHER pooled shRNA library screen identifies PP2A and FGFR signaling as potential therapeutic targets for diffuse intrinsic pontine gliomas. *Neuro Oncol.* 2019;21(7):867-877.

13. Gibson EG, Campagne O, Selvo NS, Gajjar A, Stewart CF. Population pharmacokinetic analysis of crizotinib in children with progressive/recurrent high-grade and diffuse intrinsic pontine gliomas. *Cancer Chemother Pharmacol.* 2021;88(6):1009-1020.

14. Feng Y, Xu Q, Fang M, Hu C. Anlotinib combined with temozolomide for the treatment of patients with diffuse midline glioma: a case report and literature review. *Transl Cancer Res.* 2022;11(10):3876-3882.

15. Wang Q, Niu W, Pan H. Targeted therapy with anlotinib for a H3K27M mutation diffuse midline glioma patient with PDGFR-alpha mutation: a case report. *Acta Neurochir (Wien).* 2022;164(8):2063-2066.

16. Broniscer A, Baker SD, Wetmore C, et al. Phase I trial, pharmacokinetics, and pharmacodynamics of vandetanib and dasatinib in children with newly diagnosed diffuse intrinsic pontine glioma. *Clin Cancer Res.* 2013;19(11):3050-3058.

17. Broniscer A, Baker JN, Tagen M, et al. Phase I study of vandetanib during and after radiotherapy in children with diffuse intrinsic pontine glioma. *J Clin Oncol.* 2010;28(31):4762-4768.

18. Carvalho DM, Richardson PJ, Olaciregui N, et al. Repurposing Vandetanib plus Everolimus for the Treatment of ACVR1-Mutant Diffuse Intrinsic Pontine Glioma. *Cancer Discov.* 2022;12(2):416-431.

19. Becher OJ, Hambardzumyan D, Walker TR, et al. Preclinical evaluation of radiation and perifosine in a genetically and histologically accurate model of brainstem glioma. *Cancer Res.* 2010;70(6):2548-2557.

20. Wu YL, Maachani UB, Schweitzer M, et al. Dual Inhibition of PI3K/AKT and MEK/ERK Pathways Induces Synergistic Antitumor Effects in Diffuse Intrinsic Pontine Glioma Cells. *Transl Oncol.* 2017;10(2):221-228.

21. Miyahara H, Yadavilli S, Natsumeda M, et al. The dual mTOR kinase inhibitor TAK228 inhibits tumorigenicity and enhances radiosensitization in diffuse intrinsic pontine glioma. *Cancer Lett.* 2017;400:110-116.

22. Flannery PC, DeSisto JA, Amani V, et al. Preclinical analysis of MTOR complex 1/2 inhibition in diffuse intrinsic pontine glioma. *Oncol Rep.* 2018;39(2):455-464.
